# Segmental neurofibromatosis type 2: discriminating two hit from four hit in a patient presenting multiple schwannomas confined to one limb

**DOI:** 10.1186/s12920-015-0076-2

**Published:** 2015-01-24

**Authors:** Elisabeth Castellanos, Isabel Bielsa, Cristina Carrato, Imma Rosas, Ares Solanes, Cristina Hostalot, Emilio Amilibia, José Prades, Francesc Roca-Ribas, Conxi Lázaro, Ignacio Blanco, Eduard Serra, on behalf of the NF2 Multidisciplinary Clinics HUGTiP-ICO-IMPPC

**Affiliations:** Institute of Predictive and Personalized Medicine of Cancer (IMPPC), Badalona, Spain; Department of Dermatology, Germans Trias i Pujol Hospital (HUGTiP), Badalona, Spain; Department of Pathology, HUGTiP, Badalona, Spain; Program on Hereditary Cancer, Catalan Institute of Onclogy (ICO), Badalona, Spain; Department of Neurosurgery, HUGTiP, Badalona, Spain; Department of Otorrinolaringology, HUGTiP, Badalona, Spain; Program on Hereditary Cancer, ICO-IDIBELL, Hospitalet de Llobregat, Spain; Program on Clinical Genetics and Genetic Counseling, HUGTiP, Badalona, Spain

**Keywords:** Neurofibromatosis type 2, Schwannomatosis, Multiple schwannomas, Segmental mosaicism, Genetic diagnostics

## Abstract

**Background:**

A clinical overlap exists between mosaic Neurofibromatosis Type 2 and sporadic Schwannomatosis conditions. In these cases a molecular analysis of tumors is recommended for a proper genetic diagnostics. This analysis is challenged by the fact that schwannomas in both conditions bear a somatic double inactivation of the *NF2* gene. However, SMARCB1-associated schwannomas follow a four-hit, three-step model, in which both alleles of *SMARCB1* and *NF2* genes are inactivated in the tumor, with one of the steps being always the loss of a big part of chromosome 22 involving both loci.

**Case presentation:**

Here we report a 36-year-old woman who only presented multiple subcutaneous schwannomas on her right leg. To help discriminate between both possible diagnoses, an exhaustive molecular genetic and genomic analysis was performed on two schwannomas of the patient, consisting in cDNA and DNA sequencing, MLPA, microsatellite multiplex PCR and SNP-array analyses. The loss of a big part of chromosome 22 (22q12.1q13.33) was identified in both tumors. However, this loss involved the *NF2* but not the *SMARCB1* locus. SNP-array analysis revealed the presence of the same deletion breakpoint in both schwannomas, indicating that this alteration was actually the first *NF2* inactivating hit. In addition, a distinct *NF2* point mutation in each tumor was identified, representing independent second hits. In accordance with these results, no deletions or point mutations in the *SMARCB1* gene were identified. None of the mutations were present in the blood. Two of the patient’s children inherited chromosome 22 deleted in schwannomas of the mother, but in its wild type form.

**Conclusions:**

These results conclusively confirm the segmental mosaic NF2 nature of the clinical phenotype presented.

## Background

Schwannomas are benign tumours of the nerve sheath mainly composed by Schwann cells. Solitary schwannomas can appear sporadically in the general population but when present in multiple form they are associated to Neurofibromatosis type 2 (NF2) or to Schwannomatosis. NF2 (MIM 101000) is an autosomal-dominant cancer syndrome caused by mutations in the *NF2* gene, located on chromosome 22q12.2. NF2 has an incidence of 1 in 33.000 live births, is characterized by the presence of schwannomas, meningiomas and other tumors, and distinctively characterized by the presence of bilateral vestibular schwannomas (VS) with a nearly complete penetrance at the age of 60 [1]. Over 50% of patients are familial cases and the remaining are *de novo* cases. Schwannomatosis (MIM 162091) is another autosomal-dominant syndrome characterized by the development of multiple schwannomas [2]. Schwannomatosis is partially explained by mutations in the *SMARCB1* gene [3], located 6 Mb centromeric to the *NF2* gene at 22q11.23. Approximately 10% of patients with Schwannomatosis have a family history, while the remaining 90% have sporadic disease. About 40-50% of familial Schwannomatosis and less than 10% of sporadic patients have an identifiable *SMARCB1* mutation [4-8]. A new gene, *LZTR1*, located at 22q11.21 and centromeric to *SMARCB1* has been recently reported to be mutated in ~80% of Schwannomatosis patients negative for *SMARCB1* mutation [9].

In the absence of other characteristic NF2 manifestations, the presence/absence of bilateral VS and intra-dermal schwannomas are the main clinical criteria to differentiate Schwannomatosis from NF2 [2,8]. There is a phenotypic overlap among patients that are mosaic NF2 and patients with sporadic Schwannomatosis, consisting of the presence of multiple non-vestibular nerve schwannomas. The sensitivity of blood genetic analysis is challenged in these situations [10,11]. The histological and immunohistochemical characteristics of schwannomas in Schwannomatosis and NF2 conditions are very similar, with no single robust identifiable feature serving for diagnostic purposes [2,12]. Thus, in patients with a phenotypic overlap, the molecular genetic analysis of the *NF2* and *SMARCB1* genes in at least two anatomically distinct tumors has been the recommended approach to detect the causative mutation [2,13]. The detection of the same mutation in two independent schwannomas, followed by the presence of two distinct second hits in the other alleles, could be indicative of one of the two conditions. The molecular analysis can be difficult when a first hit involves a large region of chromosome 22. In NF2-related schwannomas, sporadic vestibular schwannomas and Schwannomatosis-related schwannomas, the *NF2* gene is commonly found inactivated in both alleles, with a high percentage of mutations revealed by loss of heterozygosity (LOH) [14,15]. However, schwannomas from *SMARCB1* positive patients follow a four-hit, three-step model of tumorigenesis [4-6] in which both alleles of *SMARCB1* and *NF2* genes are inactivated in the tumor. In addition to the constitutive *SMARCB1* mutation, a second step consists in the loss of chromosome 22q, or a segment of it, involving the two loci, followed by a somatic mutation of the remaining wild-type *NF2* allele that constitutes the third step and the four hit [2]. The four-hit, three-step model, is also present in schwannomas from LZTR1 patients, involving *LZTR1* and *NF2* genes [9].

Different studies describing patient phenotypes characterized by multiple schwannomas restricted to one side or segment of the body have been reported (e.g. [14,16-18]). Some of these studies attempted to clarify the diagnosis of these patients by genetically characterizing two or more schwanomas from single patients. Some studies were not conclusive; others suggested either NF2 or Schwannomatosis. Most of these studies were performed before having a clear picture of the four-hit, three-step model, that SMARCB1 positive patients exhibit in their schwannomas.

Here we report a woman with multiple schwannomas confined to one limb. An exhaustive molecular genetic analysis was performed in two schwannomas of the patient to identify mutations in the *NF2* and *SMARCB1* genes, including cDNA and DNA sequencing, MLPA, microsatellite multiplex PCR and SNP-array analyses, to clarify the molecular diagnostics.

## Case presentation

### Clinical findings

**Figure 1:**
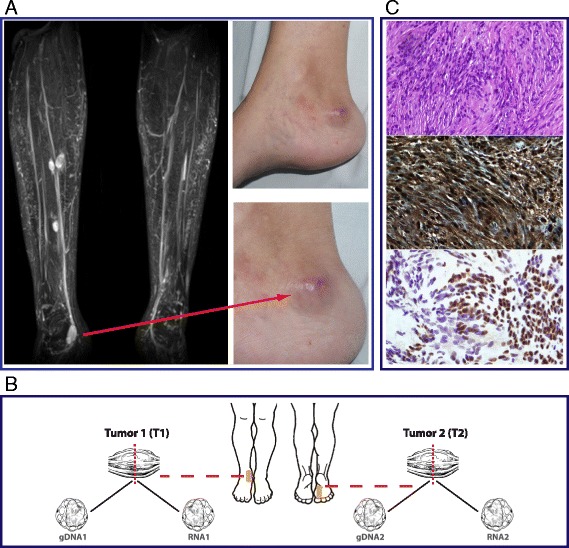
**Tumour location and description. (A)** Magnetic resonance image showing all tumors developed in patient’s right leg and picture details of one of the resected schwannomas. **(B)** Scheme of the location of the two resected schwanomas. The tumor located at the right internal malleolus (T1) and the one on the inside of the right foot (T2) were surgically removed and processed to obtain DNA and RNA from both samples. **(C)** Immunohistochemical characterization of schwannomas surgically removed. **Top image** shows that T1 was mainly composed of large spindle cells with palisaded nuclei and occasional large, hyperchromatic nuclei (degenerative atypia). In the **middle image** it can be seen that T1 tumor cells showed intense and diffuse nuclear and cytoplasmic immunoreaction for S100. **Bottom image** illustrates a SMARCB1 staining of T1 revealing a mosaic pattern of immunoreactivity.

A 36-year-old woman was referred to our NF2 Clinics because she had developed several tumors limited to the right leg during the last 6 years. Her family history was negative for cutaneous tumors or central nervous system disease. On examination, 2 deep painful tumors confined to the right calf muscle and two other violet nodules at the internal malleolus in the same leg, were palpable. General examination of the skin did not reveal cafe-au-lait spots, freckling of the axillary region, or other tumors suggestive of neurofibromas. Ophthalmological examination by split-lamp dismissed the presence of Lisch nodules, cataract or posterior lens opacities. Audiometric inspection was normal. Magnetic resonance imaging (MRI) excluded the presence of vestibular schwannomas (VS), but identified 5 tumors on the right leg (Figure 1A). The one located at the right internal malleolus (T1) and the one at the inside part of the right foot (T2) were surgically removed and molecularly analyzed (Figure 1B).

### Pathological and immunohistochemical features

Biopsy specimens from the two different tumors removed showed an encapsulated spindle cell proliferation with compact areas (Antoni A), foci of palisaded nuclei (Verocay bodies), and a less frequent loose-textured component with round nuclei and loose-knit processes (Antoni B). Focal thick-walled, hyalinized blood vessels, lymphocytic infiltrate, foamy histiocytes and degenerative tumoral atypia were also present. Strong and uniform inmunoreaction for S100 (polyclonal, dilution 1/4000, DAKO) protein was seen and analysis of *SMARCB1* expression (dilution 1/200 BD Transduction Laboratories) revealed a mosaic pattern of immunoreactivity, with patchy loss of protein expression (Figure 1C). In both cases a schwannoma was diagnosed.

### Molecular genetic analysis

**Figure 2:**
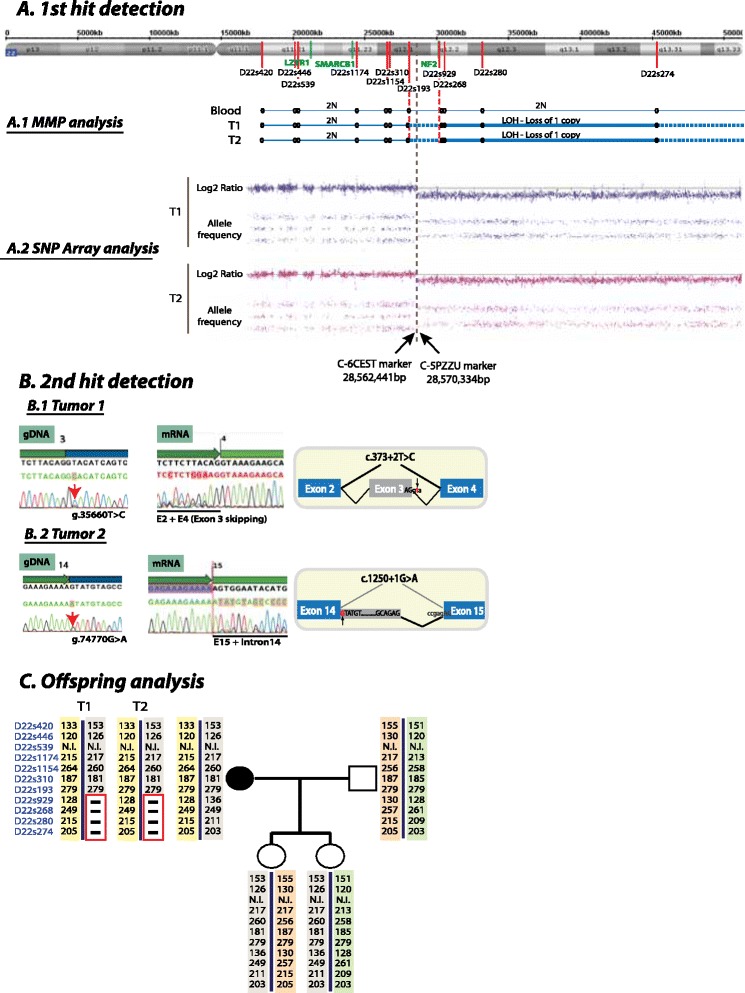
**Mutational analysis. (A)** 1^st^ hit detection: analysis of chromosome 22. **(A.1)** Chromosome 22 analysis by Multiplex Microsatellite PCR (MMP) using blood, T1 and T2 gDNA samples. Light blue line: heterozygous region; solid blue line: loss of heterozigosity (LOH); dashed line: LOH region not determined by MMP but confirmed by MLPA. Location of *NF2*, *SMARCB1* and *LZTR1* genes are indicated in green. Microsatellite position is indicated with a red line (o = no LOH, • = LOH). **(A.2)** Loss of chromosome 22 and LOH analysis characterized by SNP-array. A two-band pattern in the allele frequency plot indicates LOH from position 28570333 bp up to the telomere of chromosome 22q. Log2 Ratio < 0 indicates the loss of one copy of the same region. Breakpoint is indicated by a vertical grey dashed line. **(B)** Characterization of 2^nd^ hit *NF2* mutations in both tumors. Mutations at DNA and RNA levels are shown. The effect of mutations on splicing alteration is schematically represented. Forward sequence of exon 3 at gDNA level from T1 **(B.1)** and exon 14 from T2 **(B.2)** are shown, accompanied by the effect at mRNA level and an schematic representation. **(C)** Haplotypes of patient’s family pedigree and analyzed tumors. MMP markers are indicated in blue. Chromosome 22q loss in tumors is indicated by a red box. Patient’s daughters inherited chromosome 22 carrying the first hit mutation in patient’s tumors but in its wild-type form.

Due to the localized presence of schwannomas, a segmental mosaicism was hypothesized and so the genetic study was performed first by analyzing the two schwannomas excised. A microsatellite multiplex PCR (MMP) analysis was performed using microsatellites scattered within or around *NF2* and *SMARCB1* genes and also covering the chromosome 22q arm, using DNA from both tumors and comparing it to the blood DNA. This analysis revealed a loss of heterozygosisty (LOH) from marker D22s929 to D22s274 in both tumors, involving the *NF2* gene but not *SMARCB1* or other centromeric regions (Figure 2A). Posterior MLPA analysis (data not shown) indicated the presence of only one copy of the *NF2* gene in both tumors while the two copies of SMARCB1 were present (P258-B1 and P044-B1 kits, MRC-Holland). To characterize whether chromosome 22q deletion breakpoints were identical or not in both schwannomas, SNP-array analysis was performed (CytoScan® 750 K, Affymetrix). Both tumors revealed an identical breakpoint localized between C-6CEST (28562441 bp) and C-5PZZU (28570334 bp) markers (Figure 2A), indicating this deletion as the first hit event occurred. After that, the whole coding sequence of *NF2* and *SMARCB1* genes were sequenced in tumor T1. A homozygous g.35660 T > C change at the donor site of exon 3 (c.373 + 2 T > C) of *NF2* was identified and confirmed to be the cause of the skipping of exon 3 at mRNA level (r.254_373del; p. Lys80_Gln121del) (Figure 2B). No point mutation was identified in the *SMARCB1* gene. The *NF2* mutation g.35660 T > C found in T1 was not present in T2. In contrast, a new mutation in the donor site of exon 14 was detected (g.74770G > A; c.1250 + 1G > A), which caused the insertion of 78 bp of intron 14 at mRNA level (r.1250_1251insNG_00957.1:g.74771_074848; p.Val526Asnfs*2) (Figure 2B). Both independent mutations were interpreted as independent *NF2* second hits. None of these mutations were identified in blood, indicating the mosaic nature of the disease.

Our patient was 36 years old at the time of genetic testing and had no VS developed. Although there is risk for disease transmission of NF2 in mosaic patients [10,11] the risk level depends on the degree of mosaicism. In segmental mosaic patients the risk of disease transmission is thought to be low, but not negligible [10,11]. In our case, the study of the haplotype of the patient’s two children taken together with the results of the MLPA analysis of the mother, showed that the two children had inherited the wildtype form of chromosome 22 that is deleted in the mother; and are therefore not at risk of developing NF2.

## Conclusions

Altogether, these results indicated that the presence of schwannomas confined to one limb were caused by a first hit, the loss of one copy of 22q12.1q13.33, involving the *NF2* gene and affecting at least the schwannoma-initiating cells present in this limb segment, followed by second hits inactivating the remaining functional copy of *NF2* as an independent event in each schwannoma developed.

A minimum of 25-33% of NF2 sporadic cases are mosaic [10], and a few of them develop multiple schwannomas confined to one body segment (see for instance [14,16-18]). The four-hit, three-step model of tumorigenesis of schwannomas from *SMARCB1* positive patients forced us to perform an exhaustive mutational analysis of both *SMARCB1* and *NF2* genes, since they are somatically inactivated in both alleles. The existence of a loss of a large part of chromosome 22q in both tumors added a further complication. However, the lack of involvement of the *SMARCB1* gene (neither *LZTR1*) in the lost 22q12.1q13.33 region, together with the identification by SNP-array analysis of the same breakpoint in both schwannomas, pointed to this molecular event as the first hit occurring in at least the schwannoma-initiating cells present in this limb segment of the patient. Accordingly, two independent *NF2* mutations, one in each schwannoma, were identified as second hits. The lack of any other point mutation or small deletion in the *SMARCB1* gene in either of them, and the lack of any mutation in the blood, confirmed de NF2 segmental mosaic nature of this patient’s phenotype.

The results highlight the importance of undertaking a mutational analysis for *NF2* and *SMARCB1* in at least 2 schwannomas in sporadic patients with a Schwannomatosis phenotype before concluding a clinical diagnostics. This analysis is essential in segmental presentations. Recently, a new Schwannomatosis gene, *LZTR1*, has been discovered [9]. Schwannomas of *LZTR1* positive patients also follow a three-step four hit model involving *LZTR1* and *NF2* genes. Thus, in future studies, the molecular characterization we performed in both schwannomas will have to take into account the three genes, being guided by the extension of LOH in chromosome 22q. The identification of a first event common in all schwannomas studied allowed us to make the pre-symptomatic genetic diagnostics of the patient’s offspring. This work can contribute to incorporate genomic tools to the genetic diagnostic algorithms of hereditary cancers when mosaicism is suspected and when the analysis has to be performed preferentially at a somatic level.

### Consent

Written informed consent was obtained from the patient for publication of this Case report and any accompanying images. A copy of the written consent is available for review by the Editor of this journal.

## References

[1] Lloyd SK, Evans DG (2013). Neurofibromatosis type 2 (NF2): diagnosis and management. Handb Clin Neurol.

[2] Plotkin SR, Blakeley JO, Evans DG, Hanemann CO, Hulsebos TJ, Hunter-Schaedle K (2013). Update from the 2011 International Schwannomatosis Workshop: from genetics to diagnostic criteria. Am J Med Genet A.

[3] Hulsebos TJ, Plomp AS, Wolterman RA, Robanus-Maandag EC, Baas F, Wesseling P (2007). Germline mutation of INI1/SMARCB1 in familial schwannomatosis. Am J Hum Genet.

[4] Boyd C, Smith MJ, Kluwe L, Balogh A, Maccollin M, Plotkin SR (2008). Alterations in the SMARCB1 (INI1) tumor suppressor gene in familial schwannomatosis. Clin Genet.

[5] Hadfield KD, Newman WG, Bowers NL, Wallace A, Bolger C, Colley A (2008). Molecular characterisation of SMARCB1 and NF2 in familial and sporadic schwannomatosis. J Med Genet.

[6] Sestini R, Bacci C, Provenzano A, Genuardi M, Papi L (2008). Evidence of a four-hit mechanism involving SMARCB1 and NF2 in schwannomatosis-associated schwannomas. Hum Mutat.

[7] Rousseau G, Noguchi T, Bourdon V, Sobol H, Olschwang S (2011). SMARCB1/INI1 germline mutations contribute to 10% of sporadic schwannomatosis. BMC Neurol.

[8] Smith MJ, Wallace AJ, Bowers NL, Rustad CF, Woods CG, Leschziner GD (2012). Frequency of SMARCB1 mutations in familial and sporadic schwannomatosis. Neurogenetics.

[9] Piotrowski A, Xie J, Liu YF, Poplawski AB, Gomes AR, Madanecki P (2014). Germline loss-of-function mutations in LZTR1 predispose to an inherited disorder of multiple schwannomas. Nat Genet.

[10] Evans DG, Ramsden RT, Shenton A, Gokhale C, Bowers NL, Huson SM (2007). Mosaicism in neurofibromatosis type 2: an update of risk based on uni/bilaterality of vestibular schwannoma at presentation and sensitive mutation analysis including multiple ligation-dependent probe amplification. J Med Genet.

[11] Evans DG, Wallace A (2009). An update on age related mosaic and offspring risk in neurofibromatosis 2 (NF2). J Med Genet.

[12] Patil S, Perry A, Maccollin M, Dong S, Betensky RA, Yeh TH (2008). Immunohistochemical analysis supports a role for INI1/SMARCB1 in hereditary forms of schwannomas, but not in solitary, sporadic schwannomas. Brain Pathol.

[13] Evans DG, Raymond FL, Barwell JG, Halliday D (2011). Genetic testing and screening of individuals at risk of NF2. Clin Genet.

[14] Jacoby LB, Jones D, Davis K, Kronn D, Short MP, Gusella J (1997). Molecular analysis of the NF2 tumor-suppressor gene in schwannomatosis. Am J Hum Genet.

[15] Hadfield KD, Smith MJ, Urquhart JE, Wallace AJ, Bowers NL, King AT (2010). Rates of loss of heterozygosity and mitotic recombination in NF2 schwannomas, sporadic vestibular schwannomas and schwannomatosis schwannomas. Oncogene.

[16] Murray AJ, Hughes TA, Neal JW, Howard E, Evans DG, Harper PS (2006). A case of multiple cutaneous schwannomas; schwannomatosis or neurofibromatosis type 2?. J Neurol Neurosurg Psychiatry.

[17] Leverkus M, Kluwe L, Roll EM, Becker G, Brocker EB, Mautner VF (2003). Multiple unilateral schwannomas: segmental neurofibromatosis type 2 or schwannomatosis?. Br J Dermatol.

[18] Kaufman DL, Heinrich BS, Willett C, Perry A, Finseth F, Sobel RA (2003). Somatic instability of the NF2 gene in schwannomatosis. Arch Neurol.

